# A self‐cooling self‐humidifying mosquito carrier backpack for transporting live adult mosquitoes on foot over long distances under challenging field conditions

**DOI:** 10.1111/mve.12771

**Published:** 2024-10-26

**Authors:** Deogratius R. Kavishe, Rogath V. Msoffe, Goodluck Z. Malika, Katrina A. Walsh, Lily M. Duggan, Lucia J. Tarimo, Fidelma Butler, Emmanuel W. Kaindoa, Halfan S. Ngowo, Gerry F. Killeen

**Affiliations:** ^1^ Department of Environmental Health and Ecological Sciences Ifakara Health Institute Morogoro United Republic of Tanzania; ^2^ School of Biological Earth and Environmental Sciences University College Cork Cork Republic of Ireland; ^3^ Environmental Research Institute University College Cork, Environmental Research Institute Cork Republic of Ireland; ^4^ College of Forestry, Wildlife and Tourism, Department of Forest and Environmental Economics Sokoine University of Agriculture Morogoro Tanzania

**Keywords:** *Anopheles arabiensis*, backpack prototype, long‐distance transport, mosquito carrier

## Abstract

It is often necessary to use motorised transport to move live mosquitoes from distant field collection points into a central insectary, so that their behavioural and/or physiological phenotypes can be assessed under carefully controlled conditions. However, a survey of heritable insecticide susceptibility traits among wild‐caught *Anopheles arabiensis* mosquitoes, collected across an extensive study area composed largely of wilderness in southern Tanzania, necessitated that live mosquitoes were carried on foot over distances up to 25 km per day because most of the area was impassable by car, motorcycle or even bicycle during the rains. A self‐cooling, self‐humidifying carrier backpack was therefore developed that allows live adult mosquito specimens to be transported across rugged miombo woodland and floodplain terrain throughout the year. This wettable backpack was fabricated from stitched Tanzanian *kitenge* cotton fabric and polyvinyl chloride–coated fibreglass netting that allows easy circulation of air in and out. An outer cover flap made of cotton towelling embedded inside a kitenge envelope overhangs the fibreglass netting upper body of the bag, to protect mosquitoes from direct sunlight, and can be soaked with water to maintain low temperature and high humidity inside. Mean survival of insectary‐reared female *An. arabiensis* transported through nine different mobile camps inside the 509 km^2^ Ifakara‐Lupiro‐Mang'ula wildlife management area (ILUMA WMA), over up to 143 km and 25 days, was statistically indistinguishable from those left in the field insectary over the same period. Although considerable variance of survival was observed between different batches of mosquitoes from the insectary and between individual cups of mosquitoes, the different levels and positions inside the backpack had no influence on this outcome. Temperature and humidity inside the backpack were maintained at standard insectary conditions throughout, despite much more extreme conditions immediately outside. When the backpack was used to transport wild *An. arabiensis* and *Anopheles quadriannulatus* across a much larger study area of >4000 km^2^, encompassing the ILUMA WMA, some nearby villages and adjacent parts of Nyerere National Park (NNP), it achieved a mean survival rate of 58.2% (95% confidence interval 47.5–68.2). Encouragingly, no difference in survival was observed between ILUMA WMA and NNP even though transport back from NNP involves much longer distances, sometimes involving lengthy journeys by car or boat. Overall, this mosquito carrier backpack prototype appears to represent a viable and effective method for transporting live wild‐caught mosquitoes on foot across otherwise impassable terrain under challenging weather conditions with minimal detrimental impact on their survival.

## Graphical abstract

- A wettable backpack, made from Tanzanian kitenge and fibreglass netting, enabling year‐round transport of live mosquitoes on foot across rugged terrains.
- The backpack preserved optimal temperature and humidity levels, comparable to insectary conditions, even in harsh outdoor environments.
- Mosquito survival rates during transport were statistically comparable to those left in the insectary.

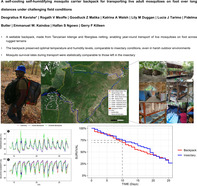

## Dhahania

Mara nyingi inahitajika kutumia usafiri wa magari kuwasafirisha mbu walio hai kutoka maeneo mbalimbali ya ukusanyaji kwenda kwenye maabara kuu, ili tabia zao ziweze kuchunguzwa katika mazingira yaliyodhibitiwa kwa uangalifu. Hata hivyo, utafiti wa sifa za kurithi zinazohusiana na usugu wa viuwadudu miongoni mwa mbu wa aina ya *Anophelesi*, waliokusanywa kwenye eneo kubwa la kusini mwa Tanzania, ulihitaji mbu wakiwa hai kusafirishwa kwa miguu hadi umbali wa kilomita 25 kwa siku kwa sababu sehemu kubwa ya eneo hilo ilikuwa haipitiki kwa gari, pikipiki au hata baiskeli wakati wa mvua. Kwa hivyo, mkoba wa kubeba mgongoni unaoweza kutunza ubaridi na unyevunyevu ulitengenezwa ili utumike kusafirisha mbu walio hai kutoka kwenye maeneo magumu ya msitu wa miombo na uwanda wa mafuriko kwa mwaka mzima. Mkoba huo uliundwa kwa kitambaa cha kitenge kilichotengenezwa kwa pamba kutoka Tanzania na wavu wenye matundu madogo madogo, ambao unaruhusu hewa kuingia na kutoka kwa urahisi. Upande wa juu wa mkoba kuna mfuniko wa kitambaa cha taulo cha pamba kilishonewa ndani ya kitenge, ambao unalinda mbu dhidi ya mwanga wa jua na unaweza kuloweshwa maji ili kudumisha hali ya ubaridi na unyevunyevu ndani ya mkoba. Mkoba huo ulipotumika kusafirisha mbu wa aina ya *Anophelesi* kutoka porini kwenye eneo kubwa la utafiti lenye zaidi ya kilomita 4000 za mraba, ulionyesha 58.2% ya mbu waliosafirishwa walibakia kuwa hai mpaka walipowasili maabara kuu. Hakukuwa na tofauti yoyote iliyoonekana kati ya mbu walisafirishwa kutoka katika hifadhi ya wanyamapori ya ILUMA na hifadhi ya taifa ya Nyerere, ingawa umbali kutoka hifadhi ya Nyerere kwenda maabara kuu ni mrefu kuliko umbali kutoka hifadhi ya ILUMA na wakati mwingine ulihusisha safari ndefu kwa gari au boti. Licha ya umbali mrefu na mazingira magumu ya usafiri, mkoba huu ulifanikisha mbu kuendelea kuwa hai hata baada ya safari ndefu, hivyo kuthibitisha uwezo wake wa kutumika kusafirisha mbu waliokamatwa kutoka sehemu zenye changamoto. Kwa ujumla, mkoba huu wa kubebea mbu umeonekana kuwa njia bora na yenye ufanisi ya kusafirisha kwa njia ya miguu mbu wa porini waliokamatwa wakiwa hai kwenye maeneo ambayo hayapitiki kwa urahisi kwa magari au pikipiki.

## INTRODUCTION

Transportation of live mosquitoes from remote field collection sites to central insectary facilities for subsequent maintenance and experimentation under controlled conditions is essential for studying their phenotypic behaviour and physiological traits. This process may be hindered by challenging terrain and weather conditions, compounded by lack of appropriate modes of transport that maximise survival of wild‐collected adult mosquitoes over the course of their journeys to a central insectary or laboratory. Wildlife conservation areas, where there may be little to no insecticide pressure, offer a unique opportunity to study mosquito populations that may serve as a refugia with natural levels of standing variation in their genomes (Rossetto & Kooyman, 2021). Such refuge populations in wild areas could, in principle, retain the full historical diversity of these species original, fully insecticide‐susceptible, wild‐type genomes, thus providing invaluable insights into natural mosquito physiology, behaviour and genetics in areas unaffected by modern insecticide use.

In order to find such refuge populations of African malaria vectors, with genomes that have not yet been bottlenecked by insecticidal selection pressure, it will probably be necessary to look beyond human‐settled areas (Epopa et al., 2020), within the many remaining well‐conserved wilderness areas that are scattered across the continent. Within the intact natural ecosystems of Africa's finest national parks, where pesticide use is negligible and wild animals offer mosquitoes diverse alternatives to humans and livestock, desirable wild‐type susceptibility traits may well also be conserved. Even on the fringes of such pristine areas, where less rigorous conservation models like community‐based wildlife management areas (WMAs) (Lee, 2018; Mwakaje, 2008) result in mixed land cover, such ecologically diverse patchworks of quite different environmental conditions may undermine the impacts of vector control (Killeen & Reed, 2018) and attenuate the selection pressures that underpin emergence of resistance traits (Mangan et al., 2023).

However, due to their typically limited infrastructure and road access, conducting entomological surveys in the most remote and pristine reaches of conservation areas can be challenging especially, when one needs to not only capture adult mosquitoes but also bring them back alive to a central insectary or laboratory for further investigation. The study reported herein therefore responds to that need, as part of a larger project intended to demonstrate that the *portfolio effects* (Killeen & Reed, 2018) caused by wildlife conservation areas allow refugia population of pyrethroid‐susceptible malaria vectors to persist, which might spread back into the nearby human‐settled area if astute insecticide combination were deployed to favour their survival (Lynch & Boots, 2016; White et al., 2014).

**FIGURE 1:**
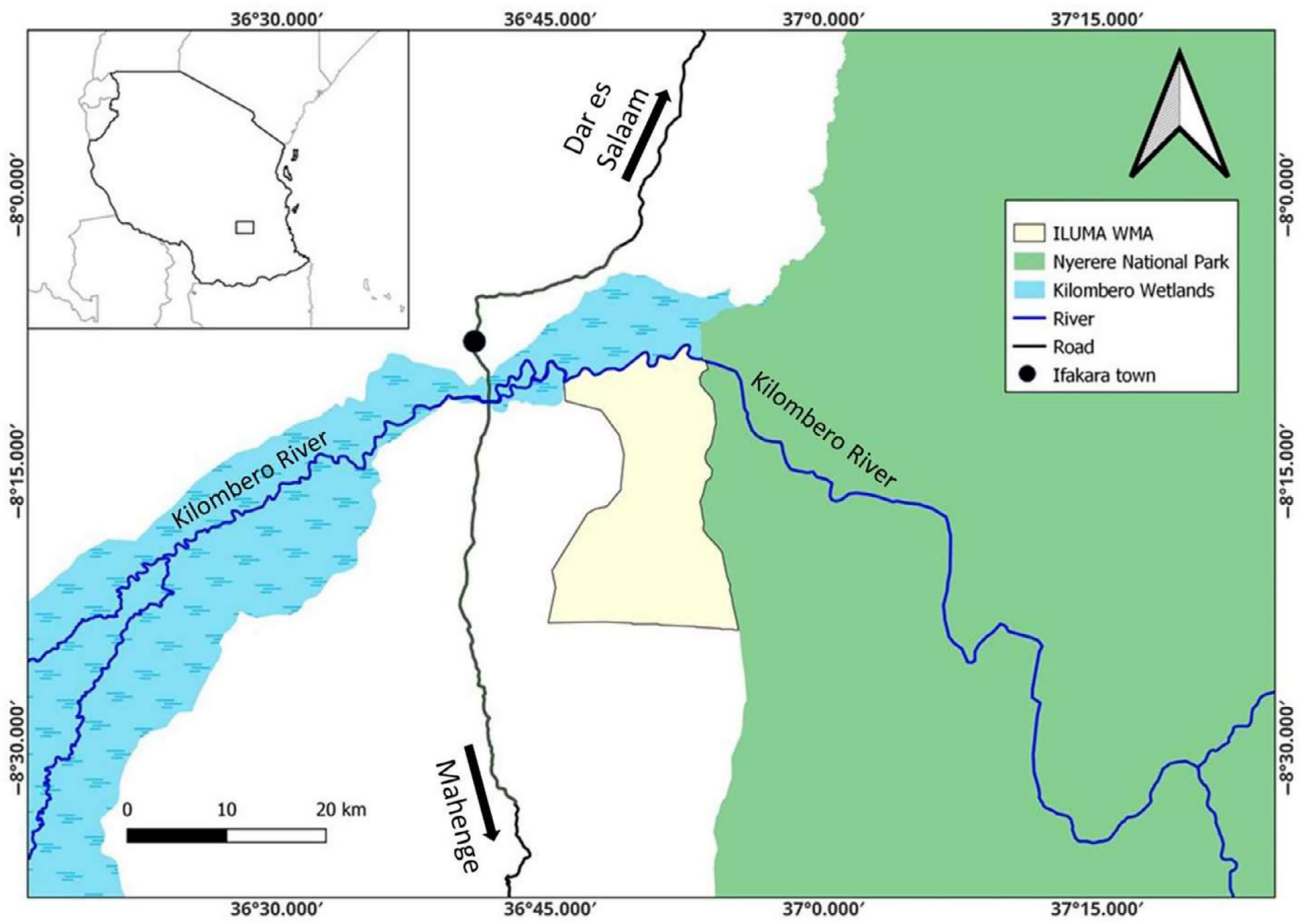
Map displaying the survey area in a national and local context in southern Tanzania. The insert at the top left is a map of Tanzania, while the enlarged area shows the part of the Ifakara‐Lupiro‐Mang'ula Wildlife Management Area (ILUMA WMA) to the south of the Kilombero River, where most of the surveys were carried out, illustrated in the context of the nearby Nyerere National Park to the east, the extensive wetlands of the Kilombero Valley inland delta upstream to the west, and Ifakara town to the north‐west.

In brief, a mosquito carrier backpack was designed and evaluated to transport live wild‐collected mosquitoes on foot. The extensive study area mosquitoes were transported across spanned a large community‐managed WMA (Lee, 2018; Mwakaje, 2008; Tourism, 2022), nearby villages, and an adjacent national park in southern Tanzania (Figure 1), most of which is otherwise completely inaccessible for much of the year (Duggan, 2023; A. K. Walsh, 2023). The aim was to bring live mosquitoes back to a central field insectary, located up to 229 km from the farthest camp, where they were used for captive propagation and further investigation of their heritable insecticide susceptibility phenotypes.

## MATERIALS AND METHODS

### Mosquito carrier backpack design

**FIGURE 2:**
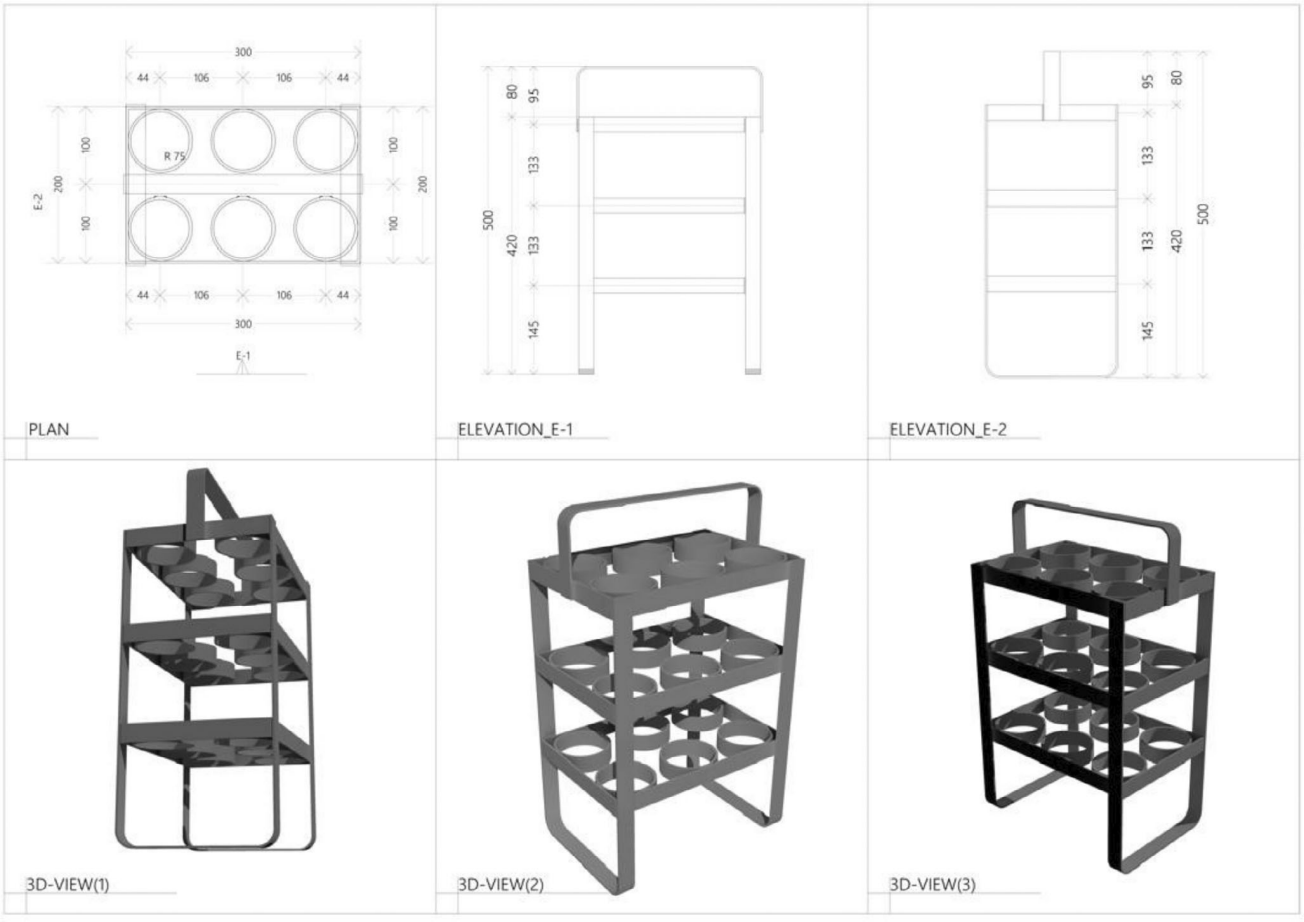
Schematic representation of dimensions of metal frame used to hold paper cups inside the mosquito carrier backpack.

**FIGURE 3:**
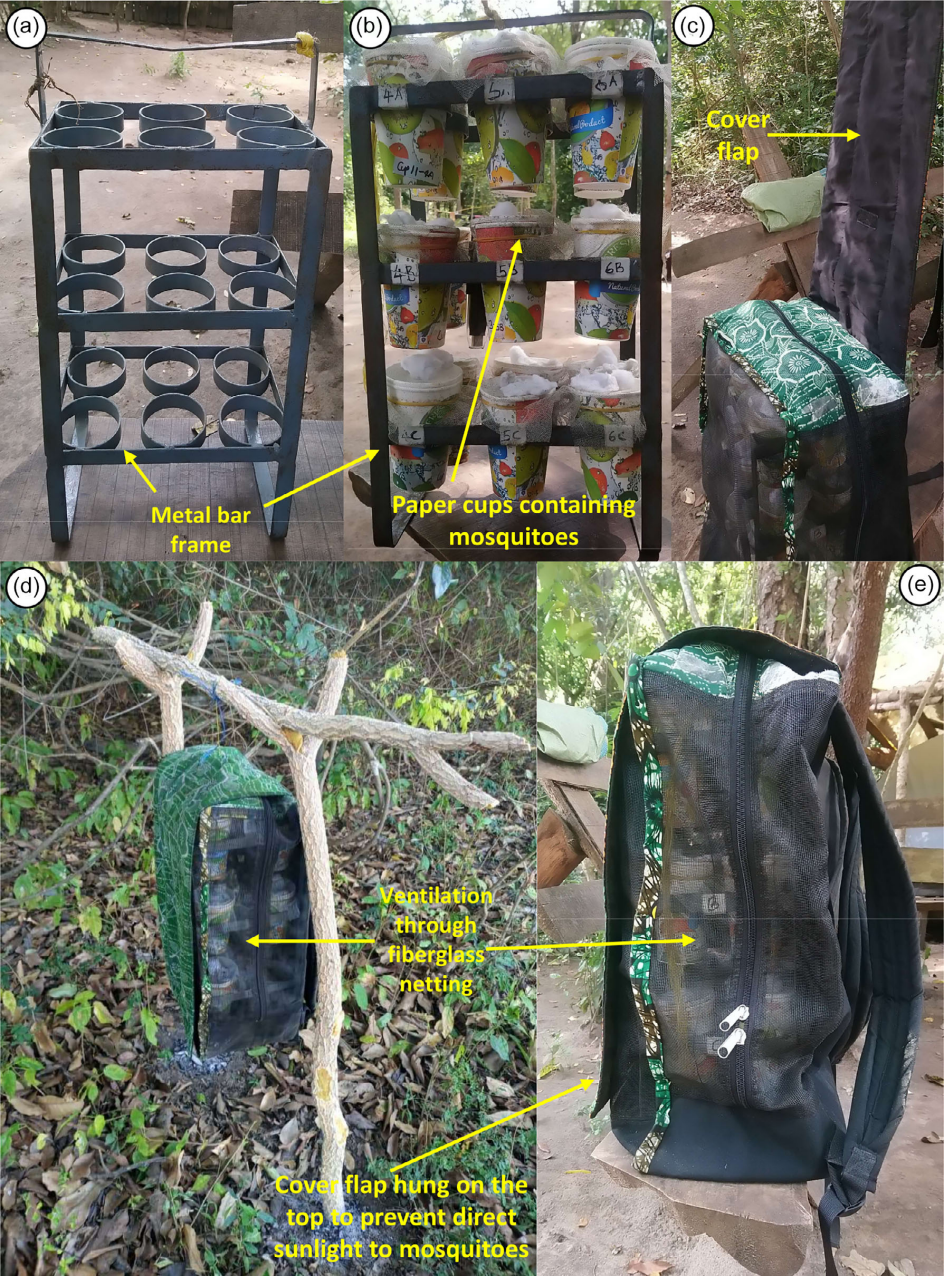
Annotated photographs of (a) the metal frame designed to hold paper cup cages of adult mosquitoes; (b) the same metal frame filled with paper cups of mosquitoes; (c) the metal frame full of cups placed inside the bespoke carrier backpack designed and evaluated herein, just as the wettable flap cover, comprised of absorbent cotton towelling enclosed within an envelope of *kitenge* cotton fabric, is dropped over the top and back of the backpack; and (d) the backpack hung in the tree to protect the mosquitoes from ground predators such ants in particular. Panel (e) shows the same backpack after the wettable flap has been dropped into place to cover the top and back of the backpack, while still leaving the cups inside fully visible and ventilated through the fibreglass netting body of the backpack.

The mosquito carrier backpack described herein (Figures 2 and 3) was designed using locally available materials, such as cotton fabric known as *kitenge*, cotton towels, polyvinyl chloride (PVC)‐coated fibreglass netting that is commonly used for window screening and a one‐inch‐wide flat metal bar. The metal bar was cut, bent and welded into the dimensions described in Figure 2, to make a metal frame with three levels that can hold six paper cups in each level (Figures 2 and 3a,b).

To make a complete mosquito carrier backpack, the metal frame was placed inside a wettable backpack with dimensions of 42 cm height × 30 cm length × 20 cm width (Figures 2 and 3a,e) tailored from cotton fabric and PVC‐coated fibreglass netting that allows easy air circulation in and out (Figure 3d,e). An outer cover flap measured 65 cm height × 30 cm length made of cotton towel embedded inside a *kitenge* fabric envelope hangs over the fibreglass netting on the upper body of the bag to protect mosquitoes from direct sunlight (Figure 3c,e). This absorbent cover flap can also be soaked with water to maintain low temperature and high humidity inside the bag. For these experiments, two mosquito carrier backpacks were made and used to transport mosquitoes.

### Study setting

This study was largely conducted at the downstream fringe of the inland delta of the Kilombero River (Figure 1) which is the largest low‐altitude wetland in east Africa, located in the Morogoro region of southern Tanzania (Mombo et al., 2011; RIS, 2002). The region has what are referred to as a short and a long rainy season, from approximately November to February and from March to April, respectively. The short rains are typically intermittent, sporadic and unreliable, whereas the long rains are usually more predictable and consistent. The dry season generally lasts from June to December, during which time the area experiences little to no rainfall. The malaria transmission system and vector populations of the Kilombero valley have been exceptionally well‐characterised (Drakeley et al., 2000; Killeen et al., 2006; Msugupakulya et al., 2023; Russell et al., 2011), and following the disappearance of *Anopheles gambiae s.s* after successful scale up of long‐lasting insecticidal nets, the two main vectors of malaria in the area are now *Anopheles funestus* Giles and *Anopheles arabiensis* Patton (Kaindoa et al., 2017; Lwetoijera et al., 2014; Maia et al., 2016; Mapua et al., 2022).

Located south‐east of Ifakara town, lies more than 500 km^2^ of community lands under the stewardships of 15 villages spanning both Kilombero and Ulanga districts which is collectively managed through the Ifakara‐Lupiro‐Mang'ula (ILUMA) WMA (Figures 1 and 3). It was formally issued a user right in 2015 and gazetted as WMA for the purpose of practicing sustainable land use and promoting wildlife conservation in the buffer zone between a rigorously conserved state‐protected area (formerly the Selous Game Reserve, now NNP), and adjacent village communities, while also creating opportunities for local communities to generate income and advance rural development (MNRT, 2022). Miombo woodland is the predominant natural land cover type across most of the ILUMA WMA, although a sizeable track of dense groundwater forest stretches along the south‐bank of the Kilombero River. On the north‐bank, floodplain grassland extends to the rainforest covered Udzungwa mountains.

### Establishment of a permanent camp and field insectary at the centre of the ILUMA WMA

**FIGURE 4:**
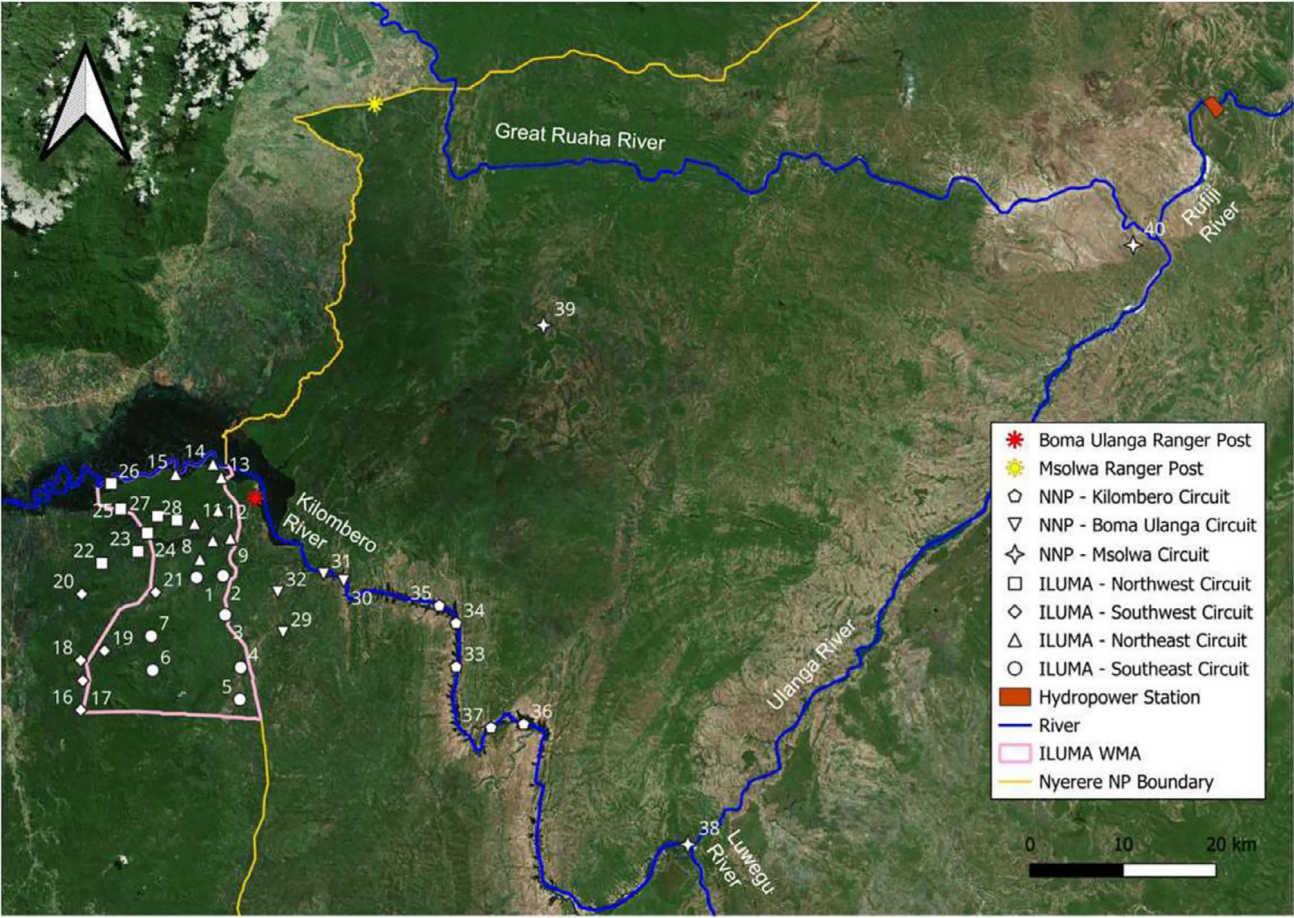
Map displaying the distribution of suitable camping locations used as the sampling frame for all the surveys described in this report. Each of the 40 camps detailed in Supplementary File 1 are illustrated in the geographic context of the boundaries of the Ifakara‐Lupiro‐Mang'ula Wildlife Management Area (ILUMA WMA) and Nyerere National Park (NNP).

**FIGURE 5:**
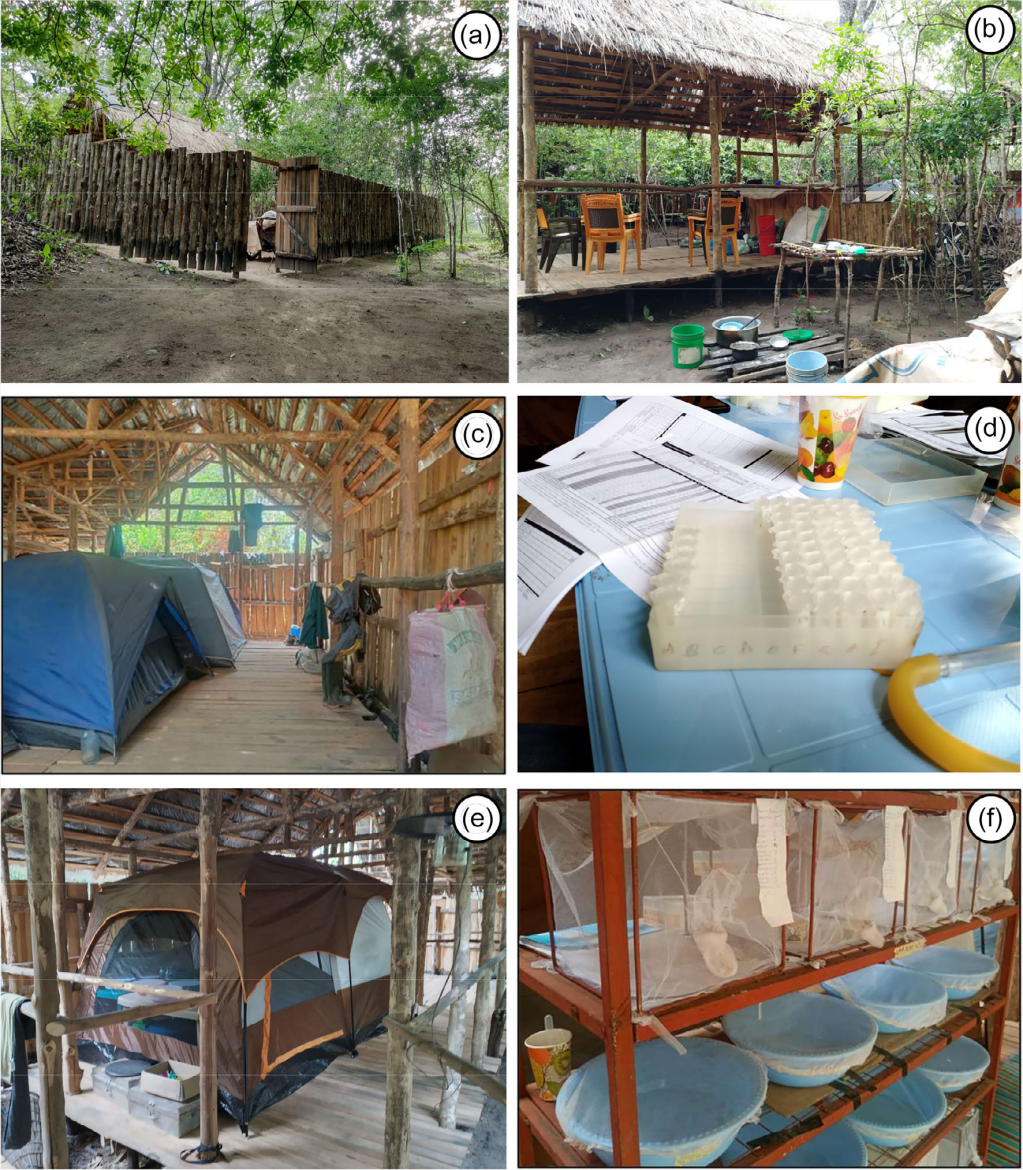
Illustrative photographs of the central camp established for the project at *Msakamba* (Figure 4). (a) The surrounding fence for security. (b) The kitchen and food storage area. (c) Tents for sleeping in. (d) An area for sorting of wild‐caught mosquitoes. (e) A field insectary tent for housing mosquito adults and larvae. (f) Mesh cages and plastic water basins for respectively rearing adult and larvae in the field insectary.

Colonised *An. arabiensis* were obtained from the IHI central insectary facility in Ifakara (Figure 1) and transferred to a field insectary established at the central camp for this project at *Msakamba* (Camp 1 in Figure 4 and Supplementary File 1), where they were propagated as per standard procedures to provide offsprings for evaluating the mosquito carrier backpack described above. The *Msakamba* camp, which is named after the seasonal stream it was built alongside, was established as a logistical operations hub for the overall project that this study was nested within, the overall goal of which was to establish whether wild‐caught *An. arabiensis* malaria vector mosquitoes collected from locations where humans and their livestock are scarce or absent may retain insecticide susceptibility traits that have been lost from mosquito populations in nearby towns and villages (Pinda et al., 2020; Urio et al., 2022). The camp was fenced and equipped with essential basic infrastructure like thatch roofs, tables, chairs, large tents, a kitchen and solar‐powered electricity supply (Figure 5).

Because most of the area within ILUMA WMA cannot be traversed by vehicle for large part of the year, the central location of *Msakamba* (Camp 1 in Figure 4 and Supplementary File 1) made it possible to reach it on foot from any other camp in ILUMA WMA within a day, so that live adult and larvae samples could usually be brought there within 48 h of collection, regardless of the weather conditions. It should be noted, however, that this sometimes‐required arduous all‐day hikes of up to 25 km to circumnavigate extensive flooded valleys. It also ensured that food supplies, as well as recharged batteries and power packs for mobile phones and field equipment, could be regularly delivered to the mobile field team, who moved from camp to camp every 2 days, along circuits of up to eight camps at a time that typically took about 2 weeks to survey (Duggan, 2023; A. K. Walsh, 2023). Village game scouts (VGS) recruited from the stakeholder communities, who are responsible for patrolling the ILUMA WMA and escorting any visitors to the conservation area, were also engaged as essential team members for this study (Duggan, 2023; A. K. Walsh, 2023). *Msakamba* was occupied and maintained on a permanent basis throughout the study, by a team of VGS and technicians, who were responsible for maintaining the field insectary, rearing field‐caught mosquitoes and carrying out experimental assessments of their insecticide susceptibility phenotypes. While the original study design for the overall project included only sampling locations (referred to herein as *camps* because each survey visit involved setting up a temporary camp there for two nights) within the WMA or the neighbouring villages immediately to the west (Duggan, 2023; A. K. Walsh, 2023), it was subsequently expanded to include 12 additional camps distributed across a much larger area within NNP to the east (Figure 4).

### Experimental assessment of the backpack with insectary‐reared mosquitoes

Ten insectary‐reared female *An. arabiensis* mosquitoes were placed in each of six labelled paper cups, each with a wedding veil netting cover secured with a rubber band on top of the cup. Each cup of the mosquito used over the course of the study was labelled with a unique serial number, which matched to a data collection form with the same number for recording mortality events, escapes and other censoring events. Mosquitoes were provided with a 10% glucose solution soaked into a ball of a cotton wool that was placed on top of the netting cover.

When a new batch of six cups of mosquitoes from the insectary was added to the transportation experiment, 18 pieces of paper were each labelled with one of the 18 metal frame position numbers and each of the six cups of mosquitoes was allocated to a random position in the frame by blindly picking a piece of paper from among the 18, reading the position number, and placing the paper cup in that specific position. This was done to eliminate bias due to temperature, humidity and light which may vary slightly inside the backpack where the metal frame is placed. At the end of the first randomization, six positions out of the 18 were occupied and that set of six cups, all originating from a single batch of mosquito from *Msakamba* insectary, was considered to represent one replicate unit of the experiment.

This process of randomization without replacement was repeated approximately every 4 days, at which point the field team completed the experimental cycle of visiting one camp other than *Msakamba*, until every position within the two metal frames for the two backpacks were collectively filled with six batches of six cups per batch, with 10 mosquitoes in each cup. At the outset, an EasyLog (500‐711, EL‐USB‐2‐LCD Lascar Electronics Ltd) was also placed inside the carrier backpack to record temperature and humidity every 30 min, and it was moved through various positions within the backpack, at the top, middle and bottom of the metal frame, every experimental day. Another logger was hung immediately outside the backpack to record temperature and humidity just outside it. For each experimental replicate, with a new batch of mosquitoes from the insectary, three additional cups containing 10 mosquitoes each were treated identically except they were kept in the field insectary and maintained there under normal rearing conditions, to serve as a control group for each batch that was not transported on foot across the study area.

### Transportation and mortality monitoring of insectary reared mosquitoes

Nine different locations (Table 1) were chosen within the ILUMA WMA, as representative destinations that the field team transported different batches of insectary reared mosquitoes on foot to and from using the mosquito carrier backpack between June and August 2023. Note that this component of the study was conducted entirely in the dry season, during which time no rain fell.

**TABLE 1:** Camp names and numbers of camps visited within Ifakara‐Lupiro‐Mang'ula (ILUMA) during the experimental transportation of insectary‐reared mosquitoes around the Wildlife Management Area (WMA) in the carrier backpack illustrated in Figures 2 and 3, so that their survival under such conditions could be assessed.

Camp number^a^	Camp name	Distance from *Msakamba* main camp (km)	Total distance travelled for each batch (km)	Mosquito batch number
2	Bwawa la msiba wa Deo	3.1	143	1
10	Bwawa la Miembemi	7.8	137	2
12	Bwawa la Maya	7.9	121	3
28	Bwawa la Semka	6.5	106	4
14	Mikeregembe	12.3	93	5
7	Bwawa la Chakacheni	8.0	68	6
4	Bwawa la Nandete	10.0	68^b^	6
3	Bwawa la Nyati	5.0	68^b^	6
26	Bwawa la Mamba Luhogi	11.0	68^b^	6

*Note*: The approximate distance from the central camp *Msakamba* to each of the camps listed below and the total distance travelled by each batch (1–6) of mosquito are also indicated.

^a^ See Supplementary File 1.

^b^ Batch 6 was the last batch of insectary mosquitoes transported from *Msakamba* and it went through the rest of the camps (3, 4, and 26) together with all other batches.

The field team spent approximately four nights at each of the mobile camps visited during which time they carried out extensive hikes around the surrounding areas while carrying the backpack of mosquitoes with them. During periods spent each day in a specific camp, or while resting in the afternoons during hikes around the area surrounding the camp, the mosquito backpack was hung off the ground under the shade of a tree, on the end of a piece of string that was tied to a suitable tree branch, to keep the samples off the ground and avoid predation by ants (Figure 3d). Note also that the backpack was kept at a minimum distance of 20 m from the campfire, to avoid mortality caused by smoke. The absorbent top cover flap was soaked with water at least each morning and evening to ensure optimal environmental condition for the mosquitoes inside the backpack. The field team recorded number of dead mosquitoes, and any censoring event like escapes or removal by ants, in each cup every morning and evening. These recordings continued until no living adults remain in the cup or the study was terminated, when the last batch of mosquitoes had been transported around the WMA for 10 days. The same mortality monitoring process was applied to the control cups that were left in the field insectary at *Msakamba* and maintained under standard rearing conditions.

After four or more days, the field team returned to the main camp and rested for the night. The following morning, another batch of six paper cups, all labelled with their own new consecutive serial numbers, was added to the backpack by following the same procedures and randomization process described above. The field team then departed again to the next mobile camp and spent four nights recording mortality in each cup every morning and evening on either side of extended daily hikes.

These cycles were repeated until six replicates of batches of six cups with 10 mosquitoes each were transported over a cumulative distance ranging from 68 to 143 km over 10–25 days. Also, the corresponding three replicates of three paper cups of mosquitoes were left in the field insectary as a control group for each mosquito batch or experimental replicate for a corresponding duration. Therefore, the whole experiment consisted of 36 transported cups of mosquitoes and nine control cups, collectively spanning six different batches of mosquitoes from the field insectary.

### Practical application of the carrier backpack to maintenance and transportation of field caught mosquitoes

The overall project that the backpack described herein was designed to enable was carried out using a rolling cross‐sectional design, with four rounds of surveys encompassing a total of 40 defined camp locations across NNP, the ILUMA WMA, and the villages immediately to the west of it over 2 years (Figure 4 and Supplementary File 1). Most sampling was carried out during the wet season because rainfall generally increases *Anopheles* mosquito abundance across most localities (Charlwood et al., 1995; Gillies & De Meillon, 1969), including this one (Kavishe et al., 2024). Adult mosquito surveys, together with complimentary surveys of mosquito larvae (A. K. Walsh, 2023; K. A. Walsh et al., 2024), land cover and activities of wildlife, livestock and humans (Duggan, 2023; Duggan, Tarimo, et al., 2024), were completed sequentially at the 40 defined camp locations. This sample of surveyed locations encompassed a range of land uses and ecosystem integrity states with a wide range of mammalian species abundance and diversity (Duggan, 2023; Duggan, Tarimo, et al., 2024; Duggan, Walsh, et al., 2024; A. K. Walsh, 2023; K. A. Walsh et al., 2024) (Figure 4, Supplementary File 1).

For the original protocol, a total of 28 camps were selected after scouting potential locations in ILUMA WMA and considering the suggestions of the VGS based on their vast personal knowledge of the area. The broad geographic distribution of the camps was planned to encompass as wide of a range of ecosystem states as possible, by including all parts of ILUMA WMA and the neighbouring domesticated land to the west. However, the exact position of a camp location was ultimately determined by the requirement for perennial surface water that *Anopheles* larvae and adults could be collected from. Accessibility by foot during the wet season was also a critical factor to consider, to ensure that each camp could be safely reached, and the transport of live mosquitoes could be completed even during periods of heavy rain and flooding. The presence of one or more glades or valleys with numerous perennial waterbodies, like waterholes, ponds and streambeds near the camp, was also required.

However, no camp in the ILUMA WMA appeared to lack signs of human disturbance, and only a few remained relatively well conserved, so four new mobile camps inside NNP were added (number 29–32, Figure 4 and Supplementary File 1) to the study design. Furthermore, to obtain samples as far away from human beings, and as deep into well‐conserved ecosystems as possible, another, eight camps inside NNP were added to the end of the fourth and final round. First, an additional subset of five camps that were accessed by boat via the Kilombero river, and correspondingly named the *Kilombero circuit*, were visited (Figure 4). Pushing even further east into the park, camps 38 to 40 were accessed by vehicle along main park roads via the Msolwa ranger post and were therefore named accordingly as the *Msolwa circuit* (Figure 4).

**FIGURE 6:**
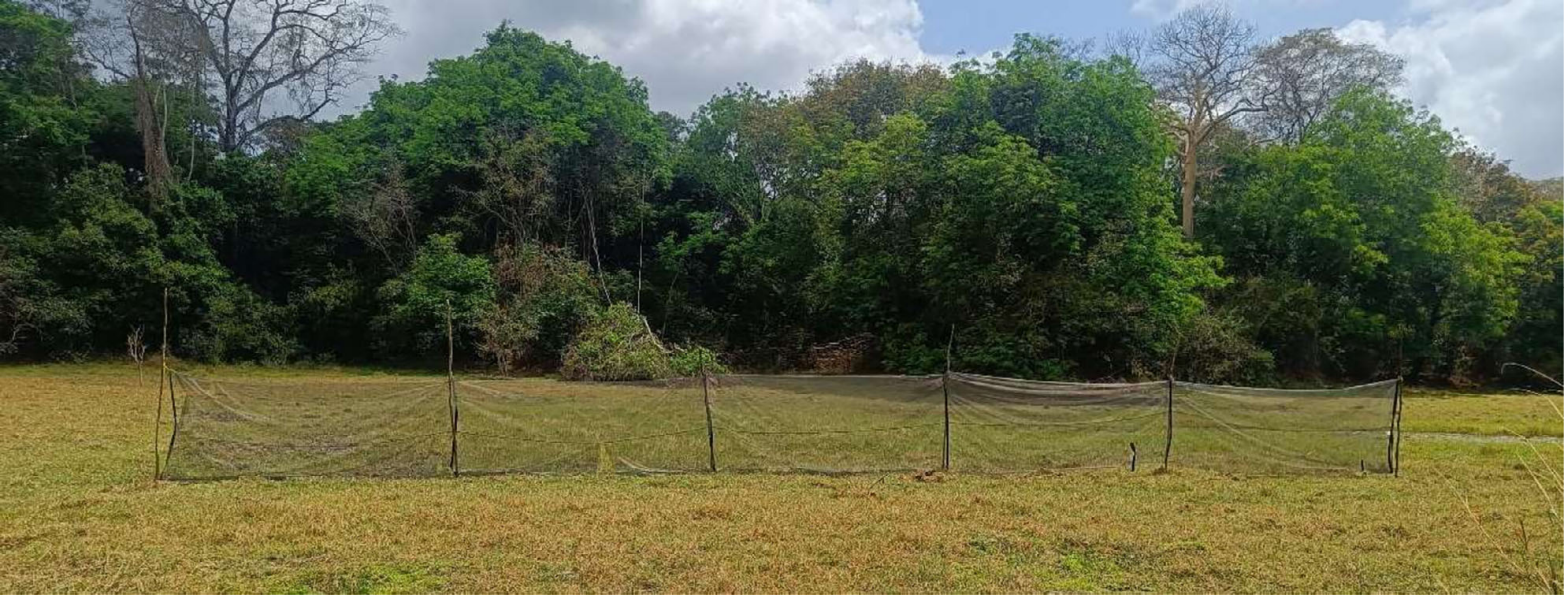
A photograph illustrating a typical set up for the screen netting interception screen trap in a typical open natural glade surrounded by dense woodland.

Live mosquitoes were trapped using two methods: a netting barrier interception screen (Figure 6) similar to that developed for sampling exophilic mosquitoes in the Pacific (Burkot et al., 2013; Davidson et al., 2018; Keven et al., 2019; Tedrow et al., 2019) and Centres of Disease and Control (CDC) light traps (John W. Hock Company, product number 512). After arriving at a new camp location, a shaded area next to a water source was scouted to identify a suitable place for setting up the tents, cooking, resting and processing mosquitoes. The batteries for four CDC light traps that were used for trapping live mosquitoes were then charged as soon as possible using portable solar panels. On overcast days, especially during the rainy season, charged batteries were received from the *Msakamba* central camp every 2 days by the team responsible for taking the collected mosquito samples. A site within a valley or open natural glade, at an approximate distance of 100 m from the camp, was identified, and a five‐panel 25‐m‐long interception screen made from standard mosquito netting was set up as shown in Figure 6.

Collections at the interception screen were carried out five times after dusk for every hour between 7:30 PM and 11:30 PM, and once just before dawn at 5:30 AM, specifically to target the known peak outdoor biting activity of adult *An. arabiensis* in the Kilombero valley (Maia et al., 2016; Russell et al., 2011). Both sides of the screen were scanned in succession, using a torch to carefully inspect each panel in an up‐and‐down motion for resting mosquitoes. Once identified, the mosquito was suctioned into a collection cup using a custom‐made, standard *Prokopack* aspirator, which is a handheld aspirator powered with a 6‐V GWESPECS battery (Maia et al., 2011; Vazquez‐Prokopec et al., 2009). Separate collection cups were used for every hour and were immediately labelled with the date and time of sampling. A small ball of cotton wool was soaked into a 10% w/v glucose solution and placed on top of the collection cup as a food source.

Additionally, four CDC light traps ran overnight between 7:00 PM and 7:00 AM, each in a different location to capture any potential variability in host‐seeking behaviour or dispersal patterns. Each trap was set up in one of four distinct ways: One suspended in an open valley or natural glade, one next to a stream bed, one in the camp site, and one next to an occupied tent. Again, the traps inside the camp and next to the tent were placed as far away from the campfire as possible. The following morning at 7:00 AM, all captured mosquitoes from the interception screen and the CDC light traps were transferred into clean, labelled paper cups using a mouth aspirator, supplied with fresh glucose solution soaked into cotton wool.

Then, an experimental design form template (ED1) from a standardised mosquito collection informatics platform (Kiware et al., 2016) was completed to include variables such as *date*, *camp number*, *collection method*, *habitat type* (referring to where the light trap or barrier screen was placed), *start time*, *finish time*, *experiment day*, *volunteer initials* and any further *comments*. Samples in the paper cups were labelled with the date and time, as well as the ED1 form *serial number*, *form row* and habitat type values, for sample processing and tracing in the field insectary at *Msakamba*. These cups containing mosquitoes were then placed back in the mosquito carrier backpack (Figure 3), following which the cover flap was soaked in water exactly as described earlier for the experimental assessment using insectary reared colony mosquitoes.

After the mosquitoes were collected and prepared on the first morning, this procedure was repeated at the same camp the following evening and morning after recharging the batteries in the afternoon. On this second morning, the field team would break camp and hike with all the equipment to the next camp location to be surveyed, but only after two VGS arriving from the *Msakamba* central camp delivered supplies and collected the backpack of mosquito samples (Figure 3e) to return them to the field insectary for sorting, counting and rearing. After arriving at the new mobile camp location, the routine described above was repeated until the relevant survey circuit was competed.

### Statistical analysis

All statistical analyses were performed by using the *R*® version 4.1.3 open‐source software package, through the *Rstudio*®, version 2023.09.1.494 environment (Team, 2022). To estimate survival of insectary‐reared female *An. arabiensis* mosquitoes, Cox regression mixed effect models were fitted to the data with the *coxme* package, treating mosquito mortality, or alternatively censoring events like escapes, predation by ants or termination of the study as the dependent response variable, while treatment (transported or non‐transported) was included as the independent variable of primary interest, whereas cage position and cup identifiers nested within insectary mosquito batch were treated as crossed random effects. Statistical contrasts between temperature and humidity measurements inside the backpack with those outside the backpack and in the insectary were accomplished by fitting generalised linear mixed models (GLMMs) using the *nlme* package with each of these outcomes treated as the Gaussian‐distributed dependent variable, location as the categorical independent variable of interest and day as standard random effect within which numbered 30‐min steps since the start of the experiment were nested to account for first order temporal autocorrelation.

Furthermore, to compare survival rates across different rounds of field collections, a logistic GLMM was fitted to the relevant observations of mosquitoes collected in the field and then transported to the *Msakamba* insectary, using the *glmer* function of the *lme4* package, treating mosquito survival as the binomial response variable, round of collection and area (ILUMA vs. NNP) as categorical fixed effect predictors, and camp number as a random effect. However, to estimate the mean survival rates for wild mosquitoes obtained with different methods of collection, an otherwise identical GLMM model but lacking an intercept was fitted to the same data, with survival as the binomial response variable, while method of collection and camp number were respectively treated as the sole categorical predictor and random effect variables.

## RESULTS

### *Experimental assessments using insectary reared* An. arabiensis

**FIGURE 7:**
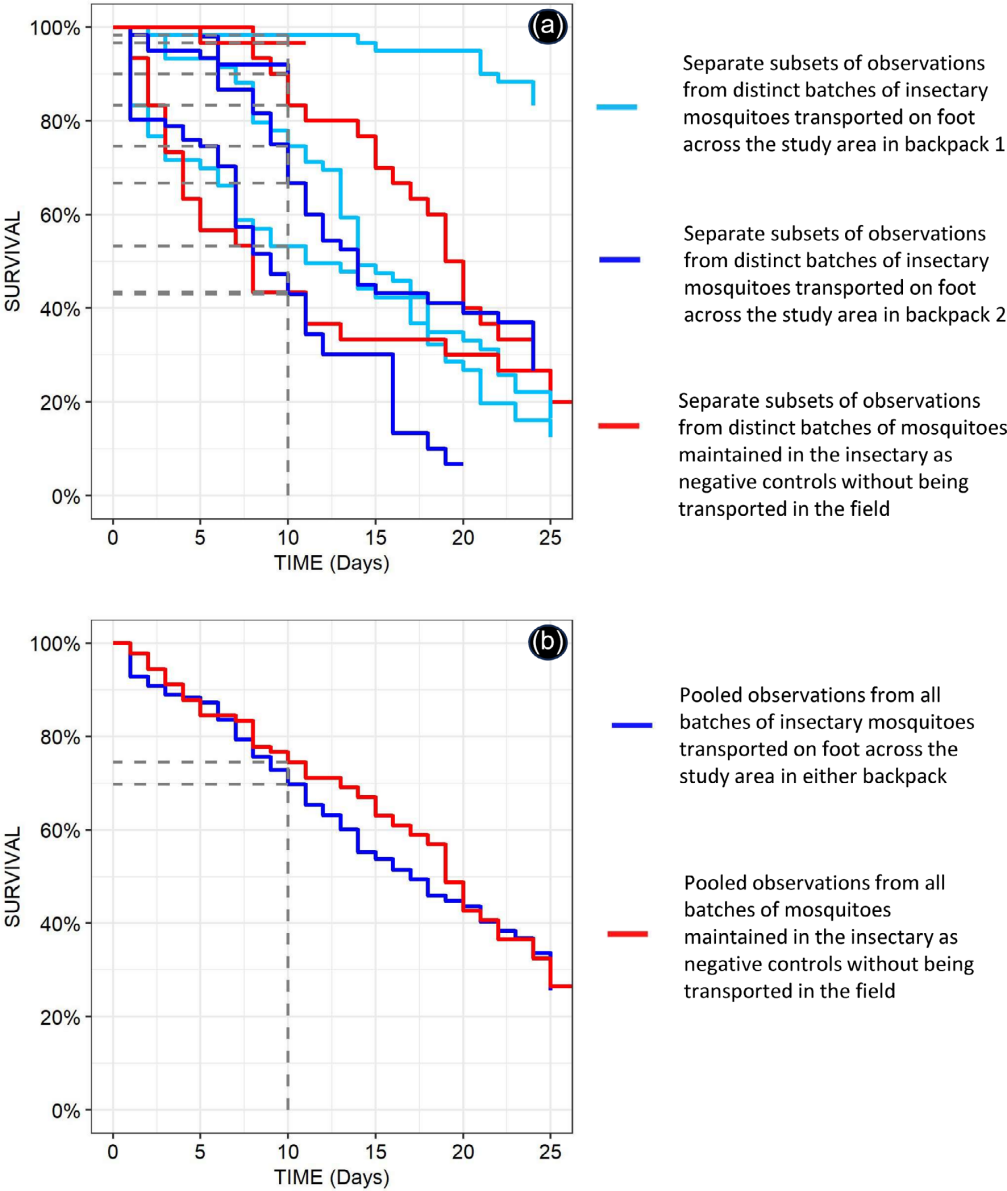
Observed survival curves from the experimentally controlled assessment, in which insectary‐reared *Anopheles arabiensis* mosquitoes were maintained inside carrier backpacks (Figure 3) over extended periods of transport on foot, compared with negative controls that were not transported but rather kept in the insectary (Figure 5e,f). Panel (a) illustrates the considerable variance between and covariance within batches of mosquitoes assigned to either long‐distance transport on foot inside the backpack or standard maintenance inside the insectary. Panel (b) illustrates how the overall mean survival curves derived from the pooled data for all the different cups and mosquito batches in the backpacks do not differ from those of the negative controls that were kept in the insectary (*p* = 0.16).

Mixed effects Cox regression, allowing for considerable covariance and variance between different batches of *An. arabiensis* colony mosquitoes from the insectary, which corresponded to quite distinctive experimental replicates in terms of their survival curves (Figure 7a), indicates that transporting mosquitoes in the backpack up to 25 days made no consistent difference (*p* = 0.16) to their overall longevity (Figure 7b). On average, across both carrier backpacks combined, at least 70% of insectary mosquitoes were still alive after 10 days of transportation. Such high survival rates over such extended periods went far beyond the original ambition of the study, which was to keep the majority of mosquitoes alive for at least 3 days in the field. While different levels and positions within the backpack had no appreciable effect upon mosquito survival (*p* = 0.20 and 0.16, respectively, for the contrasts in goodness of fit between alternative models that did and did not include these variables as random effects), considerable variation occurred between individual cups of mosquitoes (*σ* = 0.5953, *p* = 0.71 for the improvement in goodness of fit achieved by including this random effect) and between different batches of mosquitoes from the insectary (*σ* = 1.3798, *p* = 0.16 for the improvement in goodness of fit achieved by including this random effect).

**FIGURE 8:**
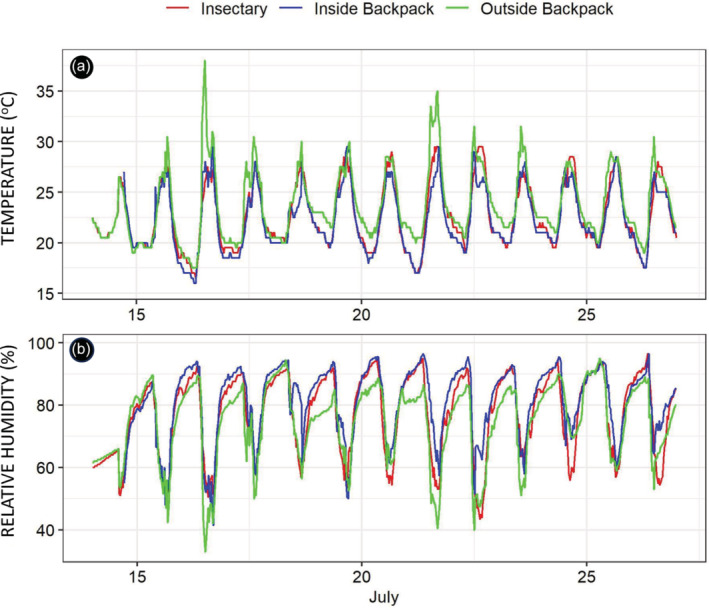
Variations in temperature (panel a) and humidity (panel b) recorded every 30 min by three data loggers over the course of 25 days and approximately 143 km of mosquito transport on foot across the study site (Table 1 and Figure 4), with one device placed inside the mosquito carrier backpack, another immediately outside mosquito carrier backpack and another inside the insectary where the negative control mosquitoes were kept over the same period.

Inside the backpacks, the temperature and relative humidity were maintained approximately within the standard recommended ranges of 17 to 27°C and 80 ± 10% respectively, for controlled rearing conditions in insectaries. Overall, temperatures were lower inside the backpack than outside it (*p* < 0.0001) or even in the insectary (*p* = 0.0122). Similarly, humidity levels were higher inside the backpack than outside it (*p* < 0.0001) or even in the insectary (*p* < 0.0001). Therefore, the original intention to develop a self‐cooling and self‐humidified mosquito carrier backpack was apparently achieved. Indeed, conditions inside the backpack while in use over long journeys, covering up to 143 km over up to 25 days, were generally very similar to those in the insectary (Figure 8). In fact, daily maximum temperatures inside the backpack were generally milder than in the insectary (Figure 8a), while humidity levels were usually the same or higher (Figure 8b). Perhaps unsurprisingly, environmental conditions inside the backpack and in the insectary were generally less harsh than in the external environment immediately outside the backpack (Figure 8).

### Practical application of the carrier backpack to maintenance and transportation of field caught mosquitoes

**FIGURE 9:**
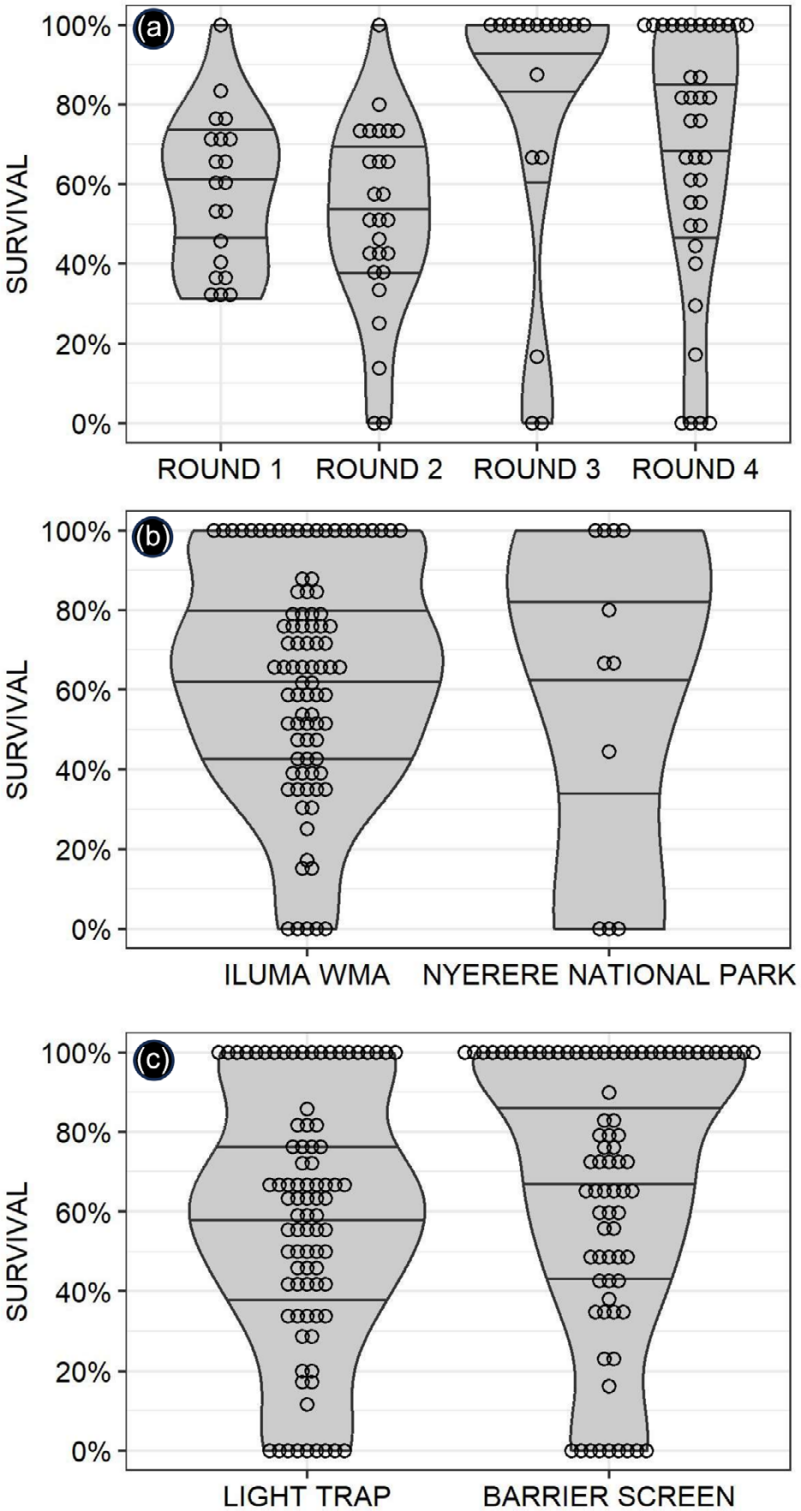
Dot plots of the distributions, medians and interquartile ranges for the observed survival rates for each two‐night collection of wild mosquitoes at each camp during each of four distinct survey rounds, over the period between being captured in the field and then maintained and transported back to the field insectary at *Msakamba* (Figure 5) from all across the study site (Figure 4) within the mosquito carrier backpack (Figure 3). (a) Comparing the four distinct rounds of surveys and associated mosquito collections. (b) Comparing mosquito collections made in or immediately to the west of the Ifakara‐Lupiro‐Mang'ula Wildlife Management Area (ILUMA WMA) and Nyerere National Park. (c) Comparing the two different mosquito capture methods used.

The overall mean (and 95% confidence interval) survival of wild‐collected *An. gambiae* complex mosquitoes, from all the scattered points of collection across the study site back to *Msakamba* (Figure 5), estimated by fitting a GLMM was 58.2% (47.5, 68.2%). Survival was somewhat higher (*p* = 0.0039) by GLMM in round three than in any of the other survey rounds (Figure 9a). This was probably because most of the troubleshooting and optimization of the field procedures for collecting mosquitoes and maintaining them in the carrier backpack had been completed by round three and the scientific team in the mobile field team remained at full strength up to that point: While there were three professional postgraduate scientists accompanying the VGS in the field throughout the first three rounds of surveys, so that the supervision of their work was distributed shared to avoid fatigue and ensure consistency, in the fourth round, this was reduced to only one. Surprisingly, no difference in survival (*p* = 0.74 by GLMM) was observed between mosquitoes collected in ILUMA WMA and those collected in NNP (Figure 9b), even though transport back from NNP involved travel over much longer distances (Figure 4), sometimes involving lengthy journeys by car or boat. Overall, mosquitoes collected from the barrier screen collection had a higher mean survival rate than those collected with CDC light traps (64.5% [58.2, 70.5%] vs. 50.0% [43.5, 56.5%], respectively, *p* ≪ 0.00001 by GLMM analysis) Figure 9c.

## DISCUSSION

This study clearly demonstrates that it is possible to transport female *An. arabiensis* mosquitoes up to 143 km on foot under challenging wilderness field conditions across a 509 km^2^ WMA for up to 25 days using a mosquito carrier backpack, with a 70% survival rate sustained consistently over 10 days. When put into practical use in a much bigger study, spanning an even larger (>4000 km^2^) and more diverse survey area (Duggan, 2023; A. K. Walsh, 2023), the majority of mosquitoes caught were successfully returned alive to the central insectary, for further investigation of their heritable insecticide susceptibility traits. This mosquito carrier backpack provided mosquitoes with favourable environmental conditions that compared well without our permanent field insectary, and it is an important advantage that it can readily made locally with affordable materials that are widely available across rural Africa. One complete unit of the mosquito carrier backpack costs a maximum of $70 to fabricate and weighs no more than 5 kg when it is fully filled with paper cups and mosquitoes, making it quite affordable, lightweight, and convenient for practical use.

Beyond addressing our project's specific needs, the backpack described herein may also have other uses. For example, the implementation of the sterile insect technique (SIT) for mosquito control always involve the transportation of live specimens from the production facility to the release points and sometimes beyond the reach of motorised transport (Ernawan et al., 2022; Gómez et al., 2022). Specifically, for SIT procedures, other factors apart from transportation such as packaging and storage conditions may affect the survival and longevity of mosquitoes (Chung et al., 2018; Sasmita et al., 2022), and any potential role for carrier backpacks based on this prototype would probably be limited to parts of remote rural areas or dense urban settlements that may be more readily accessed on foot. While transportation of sterile male *An. arabiensis* under low temperature did not significantly affect their survival (Culbert et al., 2017) and mating competitiveness (Helinski et al., 2008) over a period of up to 24 h, it may not always be access or maintain refrigeration across extensive, often remote rural areas.

To the authors' knowledge, this is possibly the only study that succeeded in transporting insectary and wild‐caught female *An. arabiensis* on foot over such long distances under such challenging field conditions, and it is encouraging that such high survival rates were achieved. Nevertheless, further experiments should be conducted to assess how well other important phenotypic characteristics, such as feeding rate, mating competitiveness, and oviposition rate are maintained after long‐distance transportation on foot using this new carrier backpack prototype.

## AUTHOR CONTRIBUTIONS

**Deogratius R. Kavishe:** Conceptualization; methodology; investigation; writing – original draft; formal analysis; project administration; supervision; data curation. **Rogath V. Msoffe:** Conceptualization; investigation; methodology; data curation. **Goodluck Z. Malika:** Validation; methodology; writing – review and editing; data curation. **Katrina A. Walsh:** Methodology; validation; writing – review and editing. **Lily M. Duggan:** Methodology; validation; writing – review and editing. **Lucia J. Tarimo:** Methodology; validation; writing – review and editing. **Fidelma Butler:** Supervision; validation; visualization; writing – review and editing. **Emmanuel W. Kaindoa:** Supervision; validation; writing – review and editing. **Halfan S. Ngowo:** Formal analysis; validation; writing – review and editing; software. **Gerry F. Killeen:** Conceptualization; investigation; funding acquisition; formal analysis; supervision; writing – review and editing; methodology; software.

## FUNDING INFORMATION

This study was primarily supported by an AXA Research Chair award to GFK, jointly funded by the AXA Research Fund in France and University College Cork (UCC) in Ireland. UCC was the primary recipient of the funds contributed by the AXA Research Fund, approximately two‐thirds of which were passed on through a subaward to the Ifakara Health Institute (IHI) in Tanzania. Institutional core funds contributed by the UCC College of Science, Engineering and Food Sciences were administered by the university's Environmental Research Institute and predominantly invested in scholarships for the Tanzanian (DRK and LJT) and Irish (KAW and LMD) postgraduate trainees involved in the project, as well as a sub‐award to Sokoine University of Agriculture in Tanzania. Supplementary funding for field equipment was kindly provided by the Irish Aid through micro‐project grant (Number IA‐TAN/2022/144) to IHI, awarded to DRK and administered by the Embassy of Ireland in Tanzania. Open access publication was funded and facilitated through the ongoing agreement between John Wiley & Sons, Inc. and the IReL consortium of Irish research libraries.

## CONFLICT OF INTEREST STATEMENT

The authors declare no conflicts of interest.

## Supporting information


**Supplementary File 1.** The number, name, location and ecological characteristics of each camp location, together with the circuit to which it was assigned and the number of times it was surveyed https://zenodo.org/doi/10.5281/zenodo.10946752.
**Supplementary File 2.** The full data set used for the analysis presented herein https://zenodo.org/doi/10.5281/zenodo.10939877.

**Data S1.** Structured reflexivity statement for international research partnerships.

## Data Availability

The data that support the findings of this study are available in the supplementary material of this article.
