# Supplementary material for: A self‐cooling self‐humidifying mosquito carrier backpack for transporting live adult mosquitoes on foot over long distances under challenging field conditions
Source: Med Vet Entomol. 2024 Oct 26;39(1):171–86. doi: 10.1111/mve.12771 (PMC11793134; doi:10.1111/mve.12771)
Supplement: Supplementary file 2 — Data S1. Structured reflexivity statement for international research partnerships. [file MVE-39-171-s001.pdf]

STRUCTURED REFLEXIVITY STATEMENT FOR INTERNATIONAL RESEARCH PARTNERSHIPS

Please complete all sections relevant to your research study

**Study conceptualisation**	
Please justify the choice to conduct the study in and/or acquire samples from the LMIC location	It is where the study was originally conceived, while in the principal investigator was based at the Ifakara Health Institute on a long-term basis as a resident collaborator.
How does this study address local research priorities and how were local researchers involved in study design?	The Ifakara Health Institute has worked on malaria as its largest priority since the institution was founded because the study setting in which it is nestled still holds the world record for measured levels of human exposure to malaria. Although the project that supported this study was originally conceived by the principal investigator who applied for the funding (GFK), as the project leader at the Ifakara Health Institute in Tanzania, I Deo Kavishe, have overseen and managed all aspects of the project, including the specific study reported in this article.
Research Management	
How has funding been used to support the local research team(s)?	As now described in the revised funding statement: "UCC was the primary recipient of the funds contributed by the AXA Research Fund, approximately two thirds of which were passed on through a subaward to the Ifakara Health Institute (IHI) in Tanzania. Institutional core funds contributed by the UCC College of Science, Engineering and Food Sciences were administered by the university's Environmental Research Institute and predominantly invested in scholarships for the Tanzanian (DRK and LJT) and Irish (KAW and LMD) postgraduate trainees involved in the project, as well as a sub-award to Sokoine University of Agriculture in Tanzania. Supplementary funding for field equipment was kindly provided by Irish Aid through micro-project grant (Number IA-TAN/2022/144) to IHI, awarded to DRK and administered by the Embassy of Ireland in Tanzania."
Data acquisition and analysis	
How are research staff who conducted data collection acknowledged?	All research staff involved in data collection are acknowledged as co-authors and have been engaged in drafting of the article.
How have members of the research partnership been provided with access to study data and analytical tools?	We have a simple rule in our collaborative team: All the data belongs to all the people who helped collect it and all are entitled to use it to generate their own research outputs that they lead as first or last authors. Thus far,

	four of the early-career researchers involved (Myself, DRK, plus LJT from Tanzanian, plus LMD and KAW from Ireland), have registered for postgraduate degrees and drafted a total of 7 first-author articles, 6 of which have already been submitted for publication.
If genetic resources were shared, how were the principles of the Nagoya Protocol on the equitable sharing of benefits	No genetic resources have been shared internationally thus far, although I am currently applying for a permit to do so, so I can visit and work with the Sanger Centre in Cambridge to apply new amplicon sequencing techniques to these samples to examine their insecticide susceptibility phenotypes.
Data interpretation	
How have research partners collaborated in interpreting study data?	All the listed authors, from all the participating institutions, have been concretely involved in the interpretation of the data and drafting of the manuscript to reflect those interpretations.
Drafting and revising for intellectual content	
How were research partners supported to develop writing skills?	I, Deo Kavishe, the corresponding author of this article drafted and then redrafted this article several times based on detailed inputs from the more experienced senior authors. I have learned a lot from this process and subsequently found it much easier to draft my second first-author article, which has already been submitted, also to Medical and Veterinary Entomology. My Tanzanian colleague at Sokoine University of Agriculture, Lucia Tarimo, has received similar support to write up complementary work on the conservation aspects of this project, while my two Irish colleagues have enjoyed similar assistance to draft and submit a total of four articles of their own.
Authorship	
How is the leadership, contribution and ownership of this work by LMIC researchers recognised within the authorship?	As the corresponding author for this article, I have drafted it as a component of my ongoing PhD thesis. My colleague and co-author, Lucia Tarimo, has also been involved with this work as part of her MSc thesis (2 first-author publications of her own forthcoming), which we are hoping will soon be extended into a full PhD.
How have early career researchers across the partnership been included within the authorship team?	All the Tanzanian (DRK and LJT) and Irish (KAW and LMD) postgraduate trainees involved in the project are listed as authors in accordance with their contributions to this

	particular article, and all have and/or will submit first-author articles of their own.
How has gender balance been addressed within the authorship?	The majority (3/4) of the contributing authors who have and/or will publish first-author publications from this project are female (KAW, LMD, LJT).
Training	
How has the project contributed to training of LMIC researchers?	As the corresponding author for this article, I have drafted it as a component of my ongoing PhD thesis. My colleague and co-author, Lucia Tarimo, has also been involved with this work as part of her MSc thesis (2 first-author publications of her own forthcoming), which we are hoping will soon be extended into a full PhD.
